# Extreme Heat Resistance of Food Borne Pathogens *Campylobacter jejuni, Escherichia coli*, and *Salmonella typhimurium* on Chicken Breast Fillet during Cooking

**DOI:** 10.1155/2012/196841

**Published:** 2012-01-29

**Authors:** Aarieke E. I. de Jong, Esther D. van Asselt, Marcel H. Zwietering, Maarten J. Nauta, Rob de Jonge

**Affiliations:** ^1^Laboratory for Zoonoses and Environmental Microbiology, National Institute for Public Health and the Environment (RIVM), 3720 BA Bilthoven, The Netherlands; ^2^Division Consumer and Safety, New Food and Consumer Product Safety Authority (nVWA), 1018 BK Amsterdam, The Netherlands; ^3^Rikilt, Institute of Food Safety, 6700 AE Wageningen, The Netherlands; ^4^Laboratory of Food Microbiology, Wageningen University, 6700 EV Wageningen, The Netherlands; ^5^National Food Institute, Technical University of Denmark, 1790 Copenhagen V, Denmark

## Abstract

The aim of this research was to determine the decimal reduction times of bacteria present on chicken fillet in boiling water. The experiments were conducted with *Campylobacter jejuni, Salmonella*, and *Escherichia coli*. Whole chicken breast fillets were inoculated with the pathogens, stored overnight (4°C), and subsequently cooked. The surface temperature reached 70°C within 30 sec and 85°C within one minute. Extremely high decimal reduction times of 1.90, 1.97, and 2.20 min were obtained for *C. jejuni, E. coli*, and *S. typhimurium*, respectively. Chicken meat and refrigerated storage before cooking enlarged the heat resistance of the food borne pathogens. Additionally, a high challenge temperature or fast heating rate contributed to the level of heat resistance. The data were used to assess the probability of illness (campylobacteriosis) due to consumption of chicken fillet as a function of cooking time. The data revealed that cooking time may be far more critical than previously assumed.

## 1. Introduction

Improper cooking is one of the main factors causing food borne illness [1–5], and a large part, 40–60%, of the cases of food borne illness are expected to originate from private households [6–9]. This is partly caused by the consumption of undercooked meat. Most consumers do not use a meat thermometer [10, 11] but determine the doneness of meat most often by cutting the meat to evaluate changes in color and texture, or by other subjective techniques. Especially for chicken breast fillet these techniques frequently result in undercooked meat [12].

Chicken breast fillet is, apart from minced meat, the most popular type of meat in The Netherlands [13] and a pathogen associated with it is *Campylobacter jejuni*, a microorganism responsible for 50% of confirmed cases of bacterial gastroenteritis in Western Europe (Austria, Belgium, Finland, France, Germany, Ireland, Italy, The Netherlands, Norway, Portugal, Sweden, Switzerland, and the UK) and the USA [1, 14–18]. Furthermore, a predominant risk factor for *C. jejuni* infection is consumption of undercooked chicken meat [19–22].

In a consumer study on food handling practices in the Netherlands by our research group [23], focusing on the preparation of a chicken breast fillet salad artificially contaminated with nonmotile, nonpathogenic *Lactobacillus casei*, it was shown that for consumers who apparently made no mistakes regarding cross-contamination, heating time of chicken meat (boiled in chicken stock) was negatively correlated with the bacterial contamination level of the prepared salad. Although this correlation may be expected, it is surprising that the bacteria present were not immediately killed upon contact with boiling water, as the bacteria were only present on the surface of the meat and not, as with ground meat, on the inside as well.

In addition, it was shown by Bergsma et al. [24] that consumer-style cooking (pan frying) of chicken breast inoculated with *C. jejuni* resulted in high bacterial heat resistance levels of this pathogen. Ample research has been dedicated to heat inactivation of bacteria in meat, but most of these studies use thin-layered ground meat as a model system and normal cooking temperatures, that is, temperatures >70°C are hardly ever applied [25–27] (also see Figure 3). As these studies do not suffice to explain the observed heat resistance by Bergsma et al. [24], the aim of this research was to further elaborate on this phenomenon. Therefore, in the current study, the chicken fillets were boiled instead of pan fried to obtain better reproducible temperatures in the experiments and decimal reduction times of bacteria present on each sample were determined. Heating profiles of chicken breast fillets during cooking were obtained both by measurement and by calculation.

Apart from *C. jejuni, Salmonella* and *Escherichia coli* were also studied as these bacteria are as well associated with food borne illness caused by consumption of raw or undercooked meat. In addition, *L. casei* was used, as this bacterium was used as indicator organism for *C. jejuni* to study real-life consumer practices by our research group [23]. Furthermore, the effect of food matrix, matrix size, and refrigerated storage on the survival of bacteria during cooking was studied as well as the effect of refrigerated storage on cell attachment. In addition, the obtained decimal reduction times were used to assess the relationship between the probability of illness (campylobacteriosis) due to consumption of chicken breast fillet and cooking time.

## 2. Materials and Methods

### 2.1. Bacterial Strains and Growth Conditions

Five *C. jejuni* strains (NCTC 11168, NCTC 11828, B258, LB99hu, and 82/69), a *Lactobacillus casei* strain (isolated from a food), and *Escherichia coli* WG5 (ATCC 700078) were used as described by De Jong et al. [28]. *C. jejuni*, *L. casei*, and *E. coli* were cultured as described by De Jong et al. [28]. *S*. *typhimurium* DT104 was grown in Brain Heart Infusion broth (37°C; BHI, Oxoid, Basingstoke, UK).

### 2.2. Study Design

The heat resistance of bacteria present on the surface of chicken breast fillets during cooking of the meat in boiling water was expressed as a decimal reduction time during boiling (*D*_boil_): time needed to reduce the number of bacteria present on the outside of a large matrix exposed to boiling water by 10-fold (min). This decimal reduction time is comparable to the formal, frequently applied *D*-value. However, to determine a *D*_*T*_-value of a bacterium on a matrix, the temperature of the matrix must be constant during the heating experiment. Therefore, *D*-values of bacteria on food items are determined using small sized food samples, mainly with a 1 mm thickness. As these small-sized samples are not representative for sizes of meats prepared by consumers during cooking, the heating experiments in this study were conducted using a normal sized meat matrix at cooking temperature. Thus, the term *D*_boil_ was introduced, with “boil” as index as the temperature could not be given explicitly, since the product temperature was dynamically changing (see Figure 2).

Furthermore, the effect of food matrix and refrigerated storage on survival of *C. jejuni* during cooking was studied. In addition, cell attachments studies were conducted. To study the matrix-effect on heat resistance of *C. jejuni*, cold-stored (4°C, 24 h) inoculated chicken breast fillets and carrots were used as matrix and survival of *C. jejuni* on these matrices after a 5 minute cooking time was compared. The effect of refrigerated storage of chicken meat on heat resistance of *C. jejuni* present on the meat surface was studied by comparing survival of *C. jejuni* present after 5 min cooking using chicken breast fillets refrigerated (4°C) for 0, 1, or 24 h. The effect of refrigerated storage on cell attachment of *C. jejuni* to the matrix was studied for both chicken breast fillet and carrot. The inoculated matrices were stored in the refrigerator (chicken meat: 0, 1, and 24 h at 4°C; carrots: 0 and 1 h at 4°C) and subsequently manually rinsed for 10 s under cold running water and the number of *C. jejuni* cells attached was determined. This was done by comparing the number of cells present on the food item after washing to the number of cells applied to the food item.

### 2.3. Inoculation Method

Food items were inoculated with a multiple-species cocktail. A multiple-species cocktail of *C. jejuni*/*L. casei* was obtained by mixing a multiple-strain cocktail (5 strains mixed in equal volumes) of *C. jejuni* with a single strain of *L. casei* in order to compare the behavior of these different bacterial species under equal test conditions, similar to what was done by the authors in a study on cross-contamination during consumer cooking practices [28]. In addition, a multiple-species cocktail containing one strain of *E. coli* and one strain *S. typhimurium* was used. The cocktails were prepared by combining equal volumes of each single-species culture [28]. An inoculum level of 10^8-9^ CFU mL^−1^ was used to inoculate the food items with. Cell counts of bacterial suspensions were determined (*N*_0_) by spread plating appropriate dilutions on appropriate media.

Heating experiments were conducted using chicken fillet or carrot. Whole chicken breast fillets (98–218 g; maximal thickness: 3.5–4 cm) purchased in different batches at a local supermarket were used fresh or were stored frozen and defrosted before use. Thawed or fresh fillets were dabbed with paper tissue to remove superficial moisture, after which each fillet was contaminated with 1 mL of inoculation culture (each side 0.5 mL), which was evenly spread using a plastic, sterile rod. Each contaminated fillet was stored overnight in a separate plastic bag at 4°C to mimic retail storage.

The (large) carrot was longitudinally sliced to obtain slices of similar weight (77–198 g) and similar thickness as the chicken breast fillets. Both sides of these slices were allowed to dry in a laminar flow cabinet (30 min each side) to remove moist, after which the carrot slices were similarly inoculated and stored as the chicken fillets.

### 2.4. Heat Treatment

For the heating experiments, one single inoculated refrigerated food item was placed in a pan (*∅* 24 cm) with boiling water (4-5 L) at a time (each data point is one single heating experiment), thus limiting the decrease of the water temperature due to the addition of the food item. The water was constantly heated and boiling. At time zero the food item was placed in the boiling water and then heated for times ranging from 0 (not heated) to 15 min. After heat treatment, the food item was immediately transferred into a sterile Waring commercial blender, weighed, and cooled with 200 mL peptone (1 g L^−1^) physiological salt (9 g L^−1^) solution (PPS) of 4°C and blended for 1 min resulting in blended chicken slurry or blended carrot slurry. The pan was washed up after cooking for every single food item and clean tap water was brought to the boil again. Experiments were conducted at least in duplicate, on different days.

### 2.5. Thermal Heat Profiles

For the heat-inactivation test, a cooking pan (*∅* 24 cm) with water (4-5 L) was brought to the boil. The water was constantly heated and as the weight of the added matrices was small (<200 g) compared to that of the water (>4000 g), the temperature profile of the water was considered to be constant at 100°C. However, the temperature of refrigerated chicken meat has a come-up time and the temperature profile of the meat can be estimated using (1):

(1) $$u_{\text{slab}}=u_{\text{chicken}\;\text{fillet}}=\frac{T_{x,t}-T_{0}}{T_{\infty }-T_{0}}=1-\mathrm{erf}\left(\frac{x}{2\sqrt{at}}\right),$$

in which *u* is the fraction of nontransferred heat (0 ≤ *u* ≤ 1), *T*_*x*,*t*_ is the temperature of the product at place coordinate *x* at time *t*, *T*_0_ is the temperature of the product at *t* = 0, *T*_*∞*_ is the temperature of the surroundings, erf is the standard error function from Excel, *x* [m] is the place coordinate, and *a* [m^2^ s^−1^] is the product's thermal diffusion coefficient, which is given by:

(2) $$a=\frac{\lambda }{\rho c_{p}},$$

where *λ* [W m^−1^ K^−1^] is the product's thermal conductivity, *ρ* [kg m^−3^] is the product's specific density, and *c*_*p*_ [Ws kg^−1^ K^−1^] is the specific heat capacity of the product, and in which only conductive heat transfer effects via the determining dimension of the chicken fillet, the height, are taken into account (the chicken fillet is thus considered to be an infinite slab, since the width and length of the fillet are more than 2.5 and 5 times as long as the height, resp.). In reality, the temperature of the chicken will increase more rapidly, so this is a fail safe assumption. Equation (1) is applicable when the external heat transfer resistance is negligible (boundary condition 1) and when the temperature of the centre of the product has not changed yet (boundary condition 2) [29].

To determine whether boundary condition 1 is met, the ratio between the transport resistance of the product and its surroundings, the Biot number (*Bi*) [29], can be calculated by:

(3) $$Bi=\frac{aL}{\lambda }=\frac{\text{transport}\;\text{resistance}\;\text{product}}{\text{transport}\;\text{resistance}\;\text{surroundings}},\;$$

where *α* [W m^−2^ K^−1^] is the convective-heat-transfer coefficient of the surroundings, *L* [m] is half height of the product, and *λ* [W m^−1^ K^−1^] the product's thermal conductivity.

When the value of *Bi* exceeds 10, the transport resistance of the surroundings is considered to be negligible. With:

(4) $$Fo=\frac{at}{L^{2}},$$

where *Fo* (Fourier number) [29] is the dimensionless time, one can calculate until which heating time, the short times boundary, boundary condition 2 is met. For an infinite slab, the short times boundary is set by *Fo* = 0.20.

The temperature profile of the chicken fillet (at different distances from the surface of the meat) in boiling water was estimated using (1) and the parameter values given in Table 1.

A PT100 temperature probe attached to an Applikon ADI-1030 biocontroller (Applikon, Schiedam, The Netherlands) was used to measure the water and surface temperature of the meat during cooking. The temperature probe was calibrated between 0°C (melting ice) and 100°C (boiling water) prior to use.

According to the manufacturer, the actual sensor is in the last two centimetres of the temperature probe. Therefore, to measure the temperature at the surface of the meat, a 20 cm long stainless steel probe was bent in the shape of an ice-hockey stick, allowing the actual sensor (last two cm) to be pressed firmly to the surface of the meat.

The temperature profiles of the water and meat surface were generated using noninoculated chicken fillets (weight: 142–172 g). The meat was placed on a plastic test tube rack so that only the last 7 cm of the temperature probe was under water. After adding the meat in the pan with boiling water, the temperature probe was immediately pressed on the meat surface or held in the water and temperature recording was started. Each experiment was repeated three times.

### 2.6. Sampling and Microbiological Enumeration

Culturability of the inoculated bacteria on the food items after heat-treatment was determined by use of the Most Probable Number method (MPN, see De Man [30]) in combination with spread-plating suitable dilutions on agar plates. Chicken breast fillets were sampled and analyzed as described by De Jong et al. [28]; the carrots were similarly treated. The lower level of detection used was 1.4 CFU per food item.

Media used for the MPN method and plate counts, respectively, were as follows, as described by De Jong et al. [28]: Preston broth and Karmali agar for *C. jejuni*, MRS broth and agar for *L. casei*, and modified-Tryptic Soy Broth and Tryptic Soy Agar both supplemented with 1 g/L nalidixic acid for *E. coli*. For *S. typhimurium* Buffered Peptone Water (NVI) and Brilliant Green Agar (Oxoid) were used. MPN samples were checked for growth of the respective organisms after incubation (time/temperature details see [28]) by streak plating on abovementioned agar plates. Suspected colonies of *C. jejuni* and *L. casei* were confirmed by phase contrast microscopy.

*C. jejuni* media were microaerobically incubated (broth: 48 h, agar: 72 h, at 37°C (to allow the recovery of any sublethally injured *C. jejuni*) either in a three-gas incubator (5% CO_2_, 10% O_2_, 85% N_2_) or in jars with BBL Campypak (Becton Dickinson, Sparks, USA). For reasons of comparison [28], media for *L. casei* were aerobically incubated at 30°C (broth 48 h; agar: 72 h), those for *E. coli* and *S. typhimurium* overnight at 37°C.

The contamination levels of the food items after heat treatment were calculated, taking into account the exact weights used for enumeration, using an Excel spreadsheet based on the MPN method described by De Man [30].

### 2.7. Data and Statistical Analysis

Data were represented as count data (log CFU and MPN per fillet) plotted versus heating time (min), to which the linear and Weibull inactivation models were fitted. Levels below the detection limit were taken into account as censored data by using maximum likelihood estimation assuming Poisson-distributed data [31]. The best fitting model was determined by applying an *F*-test using the RSS of the Weibull and linear model. Analyses were performed using Mathematica 5.2 (Wolfram Research Inc, Champaign, USA). According to the *F*-test, the linear model fitted best and therefore decimal reduction times during boiling (*D*_boil_) were calculated using the slope of the graphs, using:

(5) $$\log N_{t}=\log N_{0}-\frac{t}{D_{\text{boil}}}$$

with log as the 10-based logarithm, *N*_*t*_ as number of viable microorganisms at a given time, *N*_0_ as the initial number of microorganisms, *t* as time in min, and *D*_boil_ as time needed to reduce the number of bacteria present on the meat surface by 10-fold when exposed to boiling water.

The effect of different heat treatment scenarios was tested for its significance with ANOVA on the log transformed data in SPSS (SPSS, Chicago, USA). A significance level of 0.05 was used.

### 2.8. Comparison with Literature Data

For comparison of currently obtained data to literature data, *D*-values were collected from literature for different temperatures, strains, and products (mainly meat) or media following the approach of Van Asselt and Zwietering [32]. Microorganisms studied were *Campylobacter jejuni* (*n* = 176), *Lactobacilli* (*n* = 6), *Escherichia coli* (*n* = 79), and *Salmonella *(*n* = 287). The relation between heating temperature and log⁡*D* is linear:

(6) $$\log\;D_{\text{ref}}=\text{intercept}\;\left(\log\;D,T\right)-\frac{T_{\text{ref}}}{z}.\;$$

log⁡*D*_ref_ is the logarithm of the *D*-value (log⁡ min) at temperature *T*_ref_, *T*_ref_ is the reference temperature (°C), and *z* is the temperature increase (°C) needed to reduce the *D*-value with a factor of 10. The log⁡*D* versus temperature plot was used to compare our decimal reduction times with data (*D*-values) published in literature.

### 2.9. Risk Assessment

Decimal reduction times can be used to assess the human health risk of pathogen exposure consequential to undercooking. As an example, we studied *Campylobacter* on chicken breast fillets using an empirical distribution of concentrations on fillets after cutting at the cutting plant, obtained from a Dutch risk assessment model [21, 33]. It showed that most contaminated fillets carried between 0 between 4 log cfu/fillet and less than 5% carried higher numbers, results which are similar to *Campylobacter* contamination data reported for German retail products [34]. After incorporation of the survival after storage [21, 33], yielding a decrease in concentration described by a BetaPert distribution with minimum 0.1, most likely value 0.9 and maximum 2.1 log CFU/fillet, these results can be used as a distribution for input (*N*_0_) of (5). The exposure distribution after an inactivation time *t* is then given by the resulting distribution of *N*_*t*_. Using the same approach as Nauta et al. [35] the associated health risk, expressed as the probability of illness per fillet, can be assessed by implementing this distribution of doses into the “classic” dose response relationship for *Campylobacter *[36]:

(7) $$P_{\text{ill}}\left(N_{t}\right)=0.33\times \left(1-\frac{\Gamma \left(\alpha +\beta \right)\Gamma \left(\beta +N_{t}\right)}{\Gamma \left(\beta \right)\Gamma \left(\alpha +\beta +N_{t}\right)}\right)$$

with dose response parameters *α* and *β*.

## 3. Results

**Figure 1:**
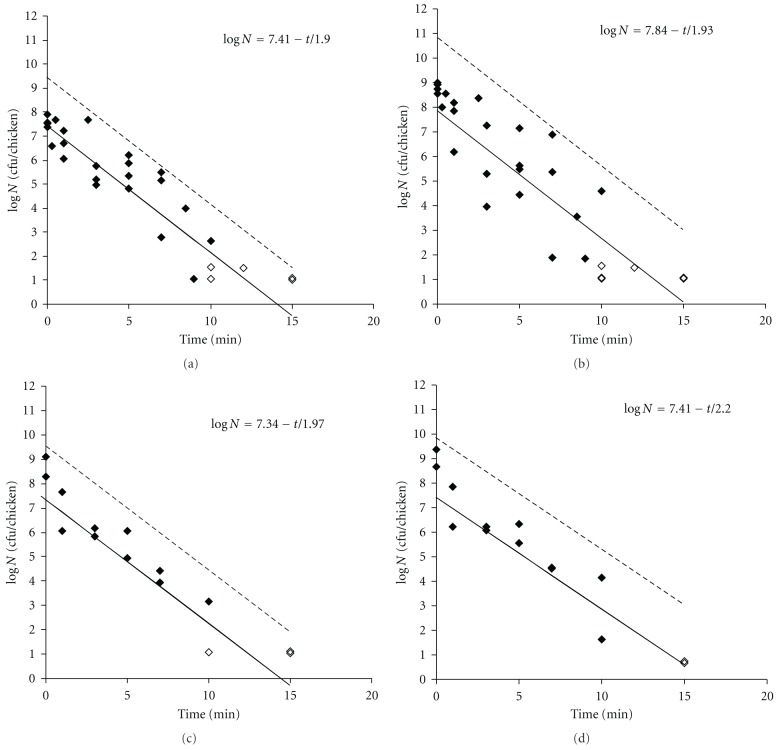
Survival of (a) *C. jejuni*, (b) *L. casei*, (c) *E. coli*, and (d) *S. typhimurium* DT104 on whole chicken breast fillets during cooking in boiling water. Straight black line: predicted model; grey line: 95% upper confidence limit; open symbols: count below detection limit.

The effect of cooking on bacterial survival on whole chicken breast fillets was studied for a cocktail of *C. jejuni.* During cooking, cell numbers declined, following a straight line (Figure 1). Using (5), we calculated that the decimal reduction time for *C. jejuni *when present on meat during cooking of the meat in boiling water was 1.90 min.

**Figure 2:**
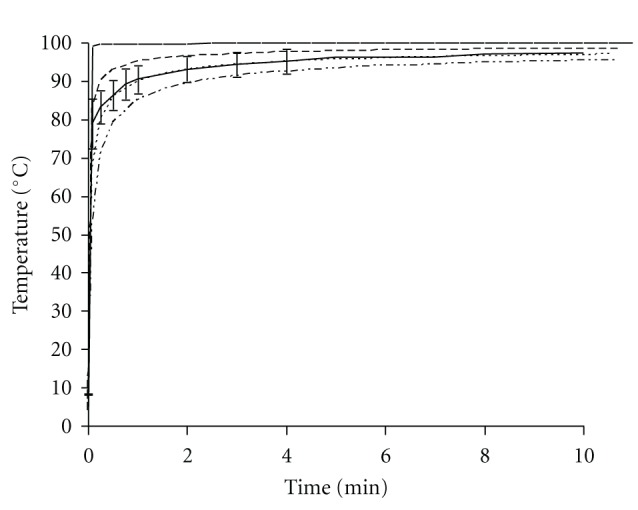
(Estimated) Temperature profiles of the surface of a chicken fillet during boiling in water. Estimated: thin —: at the surface; – –: at 0.25 mm depth; - - -: at 0.5 mm depth; –··–: at 0.75 mm depth. Measured: thick —: surface of chicken fillet including error bars for stdev.

In Figure 2, the estimated temperature profiles at the surface of a chicken fillet in boiling water and at 0.25 mm, 0.5 mm, and 0.75 mm depth are shown. To estimate the temperature profile of the chicken fillet in boiling water, two boundary conditions must be met. The first condition states that the external heat transfer resistance is negligible, thus that *Bi* > 10 (3). To verify whether this boundary condition is met, *Bi* was calculated for still water with the parameter values given in Table 1. For still water, *Bi* ranges between 11.67 and 19.33; thus for boiling water *Bi* will be higher and boundary condition 1 is met. To verify until which heating time boundary condition 2 was met, the short times boundary was calculated, using (4). We calculated a short time boundary of 10.8 min. This implies that for heating times >10.8 min the temperature according to the heating profile estimated by (1) is overestimated.

We also measured the temperature profile of the surface of the chicken fillets. After 30 sec, the average measured surface temperature of the chicken fillets approximated the estimated profile at 0.5 mm depth in the meat, and measured data ranged between the estimated profiles at 0.25 mm and 0.75 mm depth. Within 1 min, the surface temperature of chicken fillets (4°C at time zero) reached a value of at least 85°C (Figure 2). The water temperature was only slightly affected by the addition of the refrigerated meat to the water and did not drop below 99.3°C.

**Figure 3:**
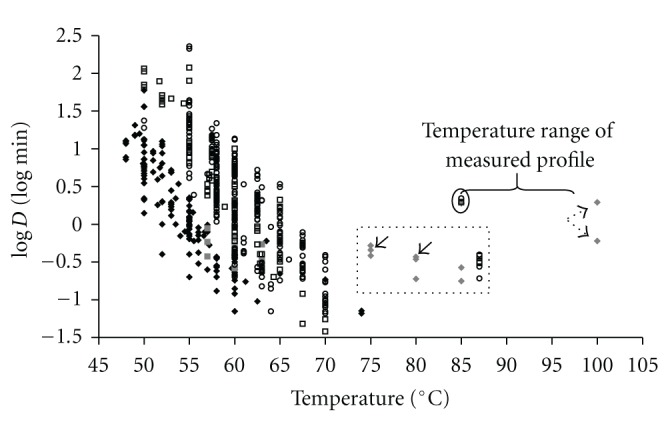
log⁡*D*-values plotted against temperature for *Campylobacter* (black diamond), Lactobacilli (grey square), *Escherichia coli* (hollow square), and *Salmonella* (hollow circle) as reported in literature. Campylobacter data for which a decimal reduction time was plotted against heating temperature, instead of *D*-value against matrix temperature: grey diamond. Encircled solid line: current *D*_boil_ data plotted against lowest surface temperature measured; }: range of the increasing temperature profile the meat was exposed to; boxed dotted line: *D*-values or decimal reduction times based on heating times ≤60 s, *T* > 70°C. Dotted arrows indicate data from Bergsma et al. [24]; solid arrows indicate data from Purnell et al. [37].

Ample research has been dedicated to heat resistance levels of food borne pathogens, and in Figure 3 an overview is given from a selection of heat inactivation data not only of *Campylobacter* but also of Lactobacilli, *E. coli,* and *Salmonella*. Data are both from fluid and solid (i.e., meat) matrices (see also Van Asselt and Zwietering [32]. This graph shows the extremity of our obtained bacterial heat resistance levels (encircled data).

The heat resistance of bacteria depends on many factors. To investigate some specificities of the observed heat resistance, different heating experiments were conducted with *C. jejuni*. In addition, the same experiment was done, but now with different bacterial species.

The heat resistance of *C. jejuni* was also determined when inoculated onto a slice of carrot of similar size as a chicken fillet to investigate whether the observed heat resistance was related to the matrix type. No bacteria were recovered from cold stored inoculated carrots (24 h, 4°C) after 5 min of boiling (data not shown).

The effect of refrigerated storage time on the heat resistance of *C. jejuni* on chicken meat was investigated. We observed that after a 24 h storage period, a 5 min cooking time reduced cell numbers by 3.0 (stdev 0.5) log units, for 1 h stored fillets this was 4.3 (stdev 0.8) log units, and for noncold stored fillets a 4.7 (stdev 0.5) log reduction was observed. The heat resistances after 0 h and 1 h storage time were not significantly different (*P* = 0.392), but those observed after a 24 h storage time were significantly higher than the other two storage times (24 h versus 1 h storage time: *P* = 0.037; 24 h versus 0 h storage time: *P* = 0.002). Cell attachment to a solid surface increases heat survival of cells [38]. Refrigerated storage for various periods of time did have no effect on the level of attachment of cells to chicken meat, as rinsing for 10 s under cold running water resulted in a 1.4–1.9 log CFU removal of Campylobacter from chicken meat, independent of the storage time (0, 1, and 24 h).

The heat resistance of *L. casei, E. coli, and S*. *typhimurium* was also tested under the same conditions as used for *Campylobacter*. For all bacterial species tested cells could still be recovered from meat after a 10 minute heating time in boiling water. Again, cell numbers declined, following a straight line. We calculated (5) decimal reduction times of 1.93, 1.97, and 2.20 min, respectively for *L. casei*, *E. coli*, and *S*. *typhimurium*.

### 3.1. Impact for Food Safety

**Figure 4:**
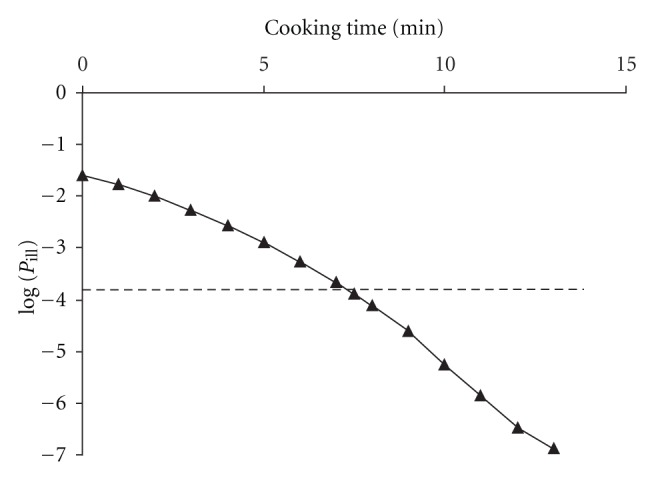
The probability of illness per consumed chicken breast fillet as a function of the cooking time. Results are obtained by a Monte Carlo simulation (400.000 runs for each cooking time *t*, given as a triangle) of (4), using the distribution of Campylobacters per fillet at retail level in The Netherlands, as found by Nauta et al. [33]. The dashed line shows the mean probability of illness per meal due to cross-contamination as found by these authors.

We finally determined the effect of the observed heat resistance levels on food safety health risks. For this purpose we applied a Dutch risk assessment study on *Campylobacter *in broiler meat, as explained in the Methods section. Equation (1) was used to calculate the expected ingested doses *N*_*t*_ after boiling the meat for *t* minutes, given the value *D*_boil_ = 1.90 min, as found in our experiments. After implementation of the dose response relationship, this resulted in Figure 4, showing the decreasing probability of illness per fillet bought at retail in The Netherlands, as a function of cooking time. Consumption of chicken breast fillets cooked for 10 minutes resulted in an estimated probability of illness of 5.5 × 10^−6^.

## 4. Discussion

This research studied the effect of consumer cooking practices on survival of various bacterial species applied to chicken fillet meat. Frying is the most frequently used method for the preparation of chicken meat by Dutch consumers [24]. The temperature at the surface of chicken meat during frying is on average 127°C, but as the temperature is difficult to control (stdev: 18°C) [24], we decided to study survival in boiling water. All tested species died off following a straight line from which a *D*-value of approximately 2 minutes was calculated. The advantage of the study design was that it immediately revealed the fate of bacteria present on meat during nonisothermal heating to high temperatures, mimicking a consumer's style of cooking. Thus, no extrapolation of data obtained from isothermal *D*- and *z*-value experiments with small-sized matrices at temperatures <70°C was needed. The disadvantage was the difficulty of determining the actual surface temperature of the meat and thus the exact temperature the bacteria were exposed to.

We measured the surface temperature and we used a mathematical approach to determine the surface temperature during cooking. With a temperature probe, we measured that the surface temperature of chicken fillets reached 85°C within 1 min. The mathematical approach can be applied when the external heat transfer resistance is negligible and when the temperature of the centre of the product has not changed. We calculated that during the first 10 minutes of cooking, both conditions are met. The calculated temperature at 0.5 mm depth in the meat approximated the average measured surface temperature of the chicken fillets. The shape of the calculated heating profile was similar to that of the measured profile, and the results of our calculations are comparable to those of Houben and Eckenhausen [39], who calculated the temperature at certain surface depths during pasteurization of a model product in a water bath of 96°C. Together this shows that both our measured and calculated temperature reliably reflect the temperature bacteria experience at the surface of a chicken breast fillet during cooking in boiling water.

Heat resistance studies are generally conducted in solid (thin patties of mainly ground meat) or in liquid matrices (broth, milk, etc.), with higher heat resistance levels obtained in solid matrices [25, 40–46]. Temperatures during such studies range from 55 to 72°C, allowing accurate determination of *D*- and *z*-values. At higher temperatures, *D*-values can only be calculated. In our experimental set-up the surface temperature of chicken fillets reached 85°C within 1 minute. According to the literature (after extrapolation) the *D*-value of bacterial cells at 85°C is less than one second [32, 41, 47], yet in our experiments it took 2 minutes to obtain a 1 log reduction of bacteria. When inoculated on another solid matrix of approximately the same size (slice of carrot) bacteria showed different behaviour. No bacteria could be detected after a heating time of 5 min. The study of Van Asselt and Zwietering [32] revealed that heat resistance can be significantly increased for certain specific matrix (high fat and low Aw)/bacterium combinations. But as both carrot and chicken meat are low fat/high Aw products, our results indicate that chicken meat itself (e.g., the presence of some specific component like iron, or an amino acid) affects the heat resistance of bacteria.

 To determine the *D*-value of a bacterial species at a certain temperature, a small volume of a bacterial culture is transferred to a relatively large volume of a preheated menstruum. In this way, the temperature come-up time is as short as possible and bacteria experience a constant temperature. As the temperature of a large and solid matrix does not come up as fast as the temperature of a small or liquid matrix, the dimension of a product can affect the heating experiment. A weak size effect on heat resistance is indeed demonstrated in a similar study conducted in our laboratory by Bergsma et al. [24], who pan fried chicken meat inoculated with *C. jejuni* using whole and diced fillets. In literature, some other heat resistance data have been published based on large size meat samples. Purnell et al. [37] observed that elevated heat resistance levels of naturally *C. jejuni* contaminated whole chicken carcasses during water immersion treatments at temperatures ≥70°C (20–30 s). The other *Campylobacter* data points in Figure 3 boxed by the dotted line were calculated using data from Whyte et al. [27]. These authors conducted hot water immersion treatments (75–80°C; 0, 10, and 20 s) with naturally and artificially contaminated chicken thighs. Again high survival levels of *Campylobacter* cells were obtained after heat treatments. In the study by Purnell et al. [37] not only was the size of the test products larger than that used in most other studies, but also the challenge temperatures in this studies were higher than normally used in heat resistance testing. This high challenge temperature might also have its effect. Interestingly, in small sized meat discs artificially inoculated with *E. coli* and *Salmonella* (see Figure 3 in dotted box) and pasteurized with hot steam (87°C), also high survival levels of bacteria were measured after a 60 s heat treatment [48]. Although the calculated *D*-values or decimal reduction times for data from Purnell, Whyte, and McCann are based on very short heating times, ≤60 s, and may therefore be less accurate, these *D*-values or decimal reduction times and our current data show that bacteria on meat are not as easily killed by temperatures >70°C as predicted based on *D*-values obtained at temperatures <70°C. Both our data and those discussed previously indicate that the combination of products size and challenge temperature might affect the level of heat resistance of bacteria.

Another difference of our study design compared to heating experiments described in literature is the overnight refrigerated storage of the contaminated fillets to mimic consumer/retail storage conditions. This could allow for physiological adaptation and attachment. Although overnight cold storage did not affect the number of bacteria attached to the meat, we observed that storing the meat in a refrigerator increased the number of surviving cells. At a low temperature (no growth, stress) physiological properties may have changed which may have affected the heat resistance of the bacteria, a phenomenon known as cross-protection [49, 50].

Although the pathogens used were all motile and thus can move from the surface to more inner parts of fillets, the observed high level of heat resistance cannot be explained by such movement, as the heat survival of the non-motile *L. casei* was equal to that of the motile pathogens tested.

So chicken meat, challenge temperature, or heating rate and cold storage have their effect on the heat resistance of *C. jejuni*, *S. typhimurium*, *E. coli,* and *L. casei. *They survive for longer periods of time than expected during cooking. Friedman et al. [51] concluded that “…because bacteria on the surface of poultry would be destroyed by limited cooking, the recurrent association of illness with undercooked poultry suggests either that the poultry is regularly re-contaminated after cooking or that *Campylobacter* is somehow present deep in the tissues of a single poultry carcass, where it survives limited cooking”, which is also in concordance with FAO/WHO conclusions [52]. However, data presented in our paper reveal that limited cooking does not necessarily eliminate all bacteria present on the surface of poultry meat. Furthermore, our data add to the deeper understanding of the frequent association of food borne illness with consumption of undercooked poultry. As a consequence, our data show that research conclusions based on the assumption that cooked chicken meat does not substantially contribute to the risk of food borne illness [53–55] must be interpreted with more consideration. Consumption of chicken meat cooked for 10 min cooking still results in a probability of illness of 5.5 × 10^−6^.

The estimated probability of illness per meal containing chicken breast fillet and a salad cross-contaminated with *C. jejuni *from the chicken fillet to the salad, as assessed in the risk assessment of Nauta et al. [21], is 1.6 × 10^−4^. This not only confirms that the risk of acquiring campylobacteriosis consequential to undercooking is much smaller than that consequential to cross-contamination [56] but also shows that the cooking time may be far more critical than previously assumed: with a cooking time of about 7.5 min the risk of undercooking is comparable to that calculated for cross-contamination (see Figure 4). Taking consumer behavior into account, using data of the observational study of Fischer et al. [23], it becomes clear that undercooking of chicken meat by consumers is certainly not negligible, as 33% of the participants (Dutch consumers) in their study applied heating times during cooking of chicken breast fillets of less than 7.5 min.

When inoculated on chicken breast fillets, the heat resistance of bacteria increased to unexpected high levels. Chicken meat, the challenge temperature, or heating rate and cold storage affected the level of resistance. It can be concluded from our study that, until now, the effect of cooking on the survival of bacteria present on the outside of chicken meat has been overestimated. It is therefore recommended to reconsider all statements made based on meat heating trials that do not use consumer-style meat types, sizes, and cooking techniques.

## Figures and Tables

**Table 1:** Parameter values for estimating the temperature profile of a chicken fillet in boiling water.

Parameter	Value
*T*_0_	4°C
*T*_*∞*_	100°C
*α*_still water_	350–580 W m^−2^ K^−1^
*L*_chicken meat_	0.02 m^*a*^
*λ*_chicken meat_	0.6 W m^−1^ K^−1^
*ρ*_chicken meat_	1192 kg m^−3^
*c*_*p*,chicken meat_	4080 Ws kg^−1^ K^−1^

^a^Maximal measured half of thickness of 10 chicken fillets.
